# Attenuation of tonic inhibition prevents chronic neurovascular impairments in a Thy1-ChR2 mouse model of repeated, mild traumatic brain injury

**DOI:** 10.7150/thno.60190

**Published:** 2021-06-16

**Authors:** James R Mester, Paolo Bazzigaluppi, Adrienne Dorr, Tina Beckett, Matthew Burke, JoAnne McLaurin, John G Sled, Bojana Stefanovic

**Affiliations:** 1 University of Toronto, Department of Medical Biophysics, Toronto, Ontario, Canada.; 2 Physical Sciences, Sunnybrook Research Institute, Toronto, Ontario, Canada.; 3 Biological Sciences, Sunnybrook Research Institute, Toronto, Ontario, Canada.; 4 Mouse Imaging Centre, The Hospital for Sick Children, Toronto, Ontario, Canada.; 5 Neuropsychiatry Program, Department of Psychiatry and Division of Neurology, Department of Medicine, Sunnybrook Health Sciences Centre, University of Toronto, Toronto, ON, Canada.; 6 Division of Cognitive Neurology, Department of Neurology, Beth Israel Deaconess Medical Center, Harvard Medical School, Boston, MA, USA.; 7 University of Toronto, Department of Laboratory Medicine and Pathology, Toronto, Ontario, Canada.

**Keywords:** Traumatic brain injury, Two-photon fluorescence microscopy, Optogenetics, Neurovascular coupling, L-655, 708

## Abstract

**Rationale:** Mild traumatic brain injury (mTBI), the most common type of brain trauma, frequently leads to chronic cognitive and neurobehavioral deficits. Intervening effectively is impeded by our poor understanding of its pathophysiological sequelae.

**Methods:** To elucidate the long-term neurovascular sequelae of mTBI, we combined optogenetics, two-photon fluorescence microscopy, and intracortical electrophysiological recordings in mice to selectively stimulate peri-contusional neurons weeks following repeated closed-head injury and probe individual vessel's function and local neuronal reactivity.

**Results:** Compared to sham-operated animals, mTBI mice showed doubled cortical venular speeds (115 ± 25%) and strongly elevated cortical venular reactivity (53 ± 17%). Concomitantly, the pericontusional neurons exhibited attenuated spontaneous activity (-57 ± 79%) and decreased reactivity (-47 ± 28%). Post-mortem immunofluorescence revealed signs of peri-contusional senescence and DNA damage, in the absence of neuronal loss or gliosis. Alteration of neuronal and vascular functioning was largely prevented by chronic, low dose, systemic administration of a GABA-A receptor inverse agonist (L-655,708), commencing 3 days following the third impact.

**Conclusions:** Our findings indicate that repeated mTBI leads to dramatic changes in the neurovascular unit function and that attenuation of tonic inhibition can prevent these alterations. The sustained disruption of the neurovascular function may underlie the concussed brain's long-term susceptibility to injury, and calls for development of better functional assays as well as of neurovascularly targeted interventions.

## Introduction

Mild traumatic brain injury (mTBI) is the most common type of traumatic brain injury globally, with an incidence of 369 in 100,000 1. Ninety percent of all TBI are mild 2, 3 and many cases of mTBI are thought to go undiagnosed 3. In addition to short-term cognitive dysfunction such as fatigue, mental clouding, and confusion, mTBI can lead to long-term neuropsychiatric and neurological deficits 4-9, with mTBI raising the probability of dementia up to 6-fold 6. Adverse effects are compounded when mTBI occurs repeatedly 10, 11. Many organizations have consequently implemented a three-strike rule in which individuals suffering mTBI for the third time are isolated from situations that previously posed risk of injury irrespectively of any symptoms 12. Clinical assessment of mTBI is confounded by the absence of contrast in the peri-contusional (proximal to injury focus) region on structural neuroimaging 13-15. Hitherto, the majority of the preclinical mTBI research effort has focused on acute neurological changes and diagnosis, leaving major gaps in our understanding of the lasting deficits after repeated mTBI. The absence of detectable neuroimaging contrast and the gaps in the understanding of chronic pathology have encumbered development of effective interventions: currently, pharmacological treatments are prescribed in the subacute and chronic stage of TBI to treat symptoms and not the root causes of dysfunction 16.

Particularly significant to understanding the lasting deficits post TBI is evaluation of the neurovascular unit, given its importance for normal brain functioning 17; and its high dynamicity in the subacute stage post injury 18. To date, studies of the neurovascular unit at the individual vessel level post mTBI have been limited to recordings at rest or *ex vivo* 19-22, leaving injury-induced changes to functional hyperemia at the single vessel level unclear. At the same time, dysregulation of functional hyperemia has been suggested as a promising therapeutic target post TBI 23. In parallel, neuronal evaluations post-mTBI have shown reduced peri-contusional firing and attenuated evoked potentials 8, 9, 24-29, and neuronal loss 20, 25, 27, 30, 31. Attenuated neuronal activity may result from differential susceptibility of excitatory *vs.* inhibitory neurons to injury 32, and in turn lead to further neurovascular unit disruptions 33. Addressing post-injury excitatory/inhibitory imbalances 8, 9, 27 has recently been proposed a means of correcting both neuronal and cerebrovascular disruptions 9. Two recent studies accordingly investigated GABA-A receptor (GABAAR) inverse agonism 26, 27. In ischemia and chronic stress, beneficial effects on functional recovery have been shown 34-36 by GABAAR antagonism via sustained, low dose administration of L-655,708, a GABAAR inverse agonist with dose-dependent specificity 37. Its applications in mTBI models have resulted in partial behavioural recovery and alleviation of post-injury increases in GABAergic tone in slice preparations 26, 27; however, the effects of GABAA receptor inverse agonism on *in situ* neurophysiological state are not known. These gaps are particularly significant given the critical role of the re-instatement of neurovascular coupling for functional recovery 17. Finally, quantitative functional assays are key to enable reliable cross-study comparisons and synthesis of results 13, 38.

Here we use simultaneous two-photon fluorescence microscopy and intracerebral electrophysiology all recordings in an optogenetic murine model of repeated mTBI to quantify the sustained effects on the neurovascular unit with low invasiveness and high spatio-temporal precision, and assess the potential for neurovascular function normalization by a disinhibitory intervention. For this study, mTBI was modeled by three closed head injuries, an intervention that has previously been shown to produce no contrast on conventional neuroimaging 39. Neurovascular readouts were quantified in the impacted tissue region (henceforth *peri-contusional tissue*). Focused photostimulation of channelrhodopsin-2 was used to probe the neurovascular unit function in light of our earlier work showing this paradigm provides more robust neuronal and vascular responses than do physiological stimuli and offers greater spatiotemporal control over the stimulation, while still providing a correlate of responses to physiological stimuli 40. We found sustained neurovascular function aberrations that parallel earlier mesoscopic scale observations in clinical populations but that have not been thus far observed in preclinical models of mTBI. The observed mTBI-induced neurovascular deficits were largely prevented via a disinhibitory treatment with L-655,708, an inverse GABAAR agonist.

## Methods

### Animals

All experimental procedures in this study followed the ARRIVE guidelines and were approved by the Animal Care Committee of the Sunnybrook Research Institute, which adheres to the Policies and Guidelines of the Canadian Council on Animal Care and meets all the requirements of the Provincial Statute of Ontario, Animals for Research Act as well as those of the Canadian Federal Health of Animals Act. Ninety-four adult (51 male and 43 female) Thy1-ChR2-YFP mice (wild-type Channelrhodopsin-2, ChR2, in pyramidal neurons 41, Jackson labs 007612, line 18), 3-6 months of age, were used in the study. Cohort sizes and sex ratios are summarized in Table 1.

### Closed head injury surgery

The induction of closed head injury was performed by adapting a previously published protocol 25. Mice were anesthetized in an induction chamber with 5% isoflurane in oxygen enriched medical air (30% O_2_, balance N_2_) and transferred to a stereotaxic frame, where they were maintained under 1.5-2% isoflurane via a nose cone. Mice were subcutaneously administered Baytril (0.02 mg/mL), Marcaine (0.1 mL), and Ringer's lactate solution (0.5-1 mL of solution with 130 mM Na, 4 mM K, 1.5 mM Ca, 109 mM Cl, 28 mM Lactate, Hospira, Canada). The scalp was shaved and following sterile surgical procedures, an incision was made in the scalp to expose the skull surrounding the impact site at +1mm ML, +0.5 mm AP. Connective tissue was removed and an impact delivered at the aforementioned location via a stereotaxic impactor (Leica Cortical Impactor, Leica, US) using the following parameters: 1.5 mm tip diameter, 2 m/s impact speed, 1 mm impact depth and 200 ms impact dwell time (corresponding to 359 kPa). Following the impact, the scalp was sutured, Ringer's lactate solution re-administered, and Betadine reapplied over the incision site. Mice were then monitored and allowed to recover in a recovery cage on a warming pad. After approximately 30 min, fully awake and alert animals were returned to their home cage in the colony. Each mouse was subjected to three cortical impacts, delivered with an inter-impact interval of 72 h. A 9% mortality rate was observed in the 47 mice that underwent impact procedures and *in vivo* experiments, due to skulls cracking in three mice as well as one incident of poor recovery from surgery. Sham animals underwent the same surgical procedures but with no impact delivery.

### Osmotic pump implant surgery

Osmotic pump implant surgeries were performed 3 days following the third closed head injury surgery. The 3-day delay to start of L-655,708 administration was chosen based on previous reports of the timing of hyper-/hypoexcitability shifts occurring in the days following CNS damage 8,9,34,35 Osmotic pumps (Alzet micro-osmotic pump Model 1002, Durect, USA) were prepared one day prior to implantation following established procedures 35. Pumps were loaded with 5 mM L-655,708 (Bio-techne, USA) in 50% DMSO/50% saline to be delivered at a dose of 200 μg/kg/day over 12 days, previously described as a dose level for GABAAR ɑ5-subunit specificity with significant effects counteracting increased tonic inhibition 35, 37, 42 in the days following injury 8, 9. The dosing was set to both significantly affect the ɑ5-subunit containing GABAARs and avoid convulsive behaviour 35, 37. Vehicle pumps were loaded with 50% DMSO/50% saline. Loaded pumps were incubated at 37°C overnight prior to surgery. On the day of the pump implantation surgery, mice were anesthetized in an induction chamber under 5% isoflurane and transferred to a stereotaxic frame with auxiliary ear bars for head fixation where they were maintained under 1.5-2% isoflurane delivered via the nose cone. Mice were given subcutaneous Baytril (0.05 mg/mL), Marcaine (0.1 mL), and Ringer's lactate solution (1 mL). The skin behind the right shoulder was shaved and cleaned with alcohol wipes and Betadine and ocular gel applied to the eyes. Following sterile surgical procedures, an incision was made behind the right shoulder and subcutaneous space was gently cleared through the incision with blunt surgical clamps. Pumps were then placed subcutaneously through the incision such that they were fully enclosed under the skin. The incision was then sutured, Betadine reapplied and another 1 mL of Ringer's lactate solution administered. Mice were allowed to recover on a warming pad in a recovery cage. When mice were awake and alert, they were returned to their home cage. Over the subsequent 48 h, the mice were monitored closely for any signs of complications due to the implant surgery.

### Surgical procedures in preparation for imaging

Mice were anesthetized with 5% isoflurane and maintained at 1.5-2-2.5% isoflurane while on a feedback-controlled temperature pad (CWE Inc., Ardmore, PA) set to 37°C. A tracheostomy was performed to enable mechanical ventilation (SAR 830/P, CWE Inc.) and a tail vein catheter placed to allow for fluorophore and anesthetic infusion. Breath rate, heart rate, arterial oxygen saturation, and pulse/breath distention were measured throughout experiments via a pulse oximeter (MouseOx, STARR Life Sciences) and recorded via an MP150 acquisition system (Biopac Systems, Canada). Representative physiological data acquisition is shown in Figure S2 and the recorded values are summarized in Table 2.

Cranial window implantation was performed following established protocols 43. Isoflurane was lowered to 1.25-1.5% and mice were head-fixed on a stereotactic frame (Narishige, Japan). Subcutaneous Ringer's lactate solution and Xylocaine (10 mg/mL, 50-100 μL volume, AstraZeneca Canada) were administered for hydration and local anesthesia, respectively. The scalp was removed and a dental drill used to create a 3-4mm craniotomy centered over the impact location, at +1 mm ML, +0.5 mm AP. The skull cap was removed; the dura was left intact unless it remained attached to the removed skull cap, and 1% agarose in phosphate-buffered saline (PBS) (Sigma-Aldrich, Canada) applied to the brain surface. A 5 mm glass coverslip for imaging only experiments or a small section of ~1 mm thick silicone-based polydimethylsiloxane (PDMS) 44 for combined imaging and electrophysiology experiments was placed over the agarose and secured with cyanoacrylate glue. Dental cement (Land Dental, USA) was then used to create an immersion well surrounding the window. Prior to imaging, a 70 kDa Texas Red dextran (25 mg/kg body weight, Invitrogen, USA) dissolved in PBS (8.33 mg/mL) was injected via a tail vein catheter.

### Two-photon fluorescence microscopy (2PFM) and focused optogenetic stimulation

**Figure 2:**
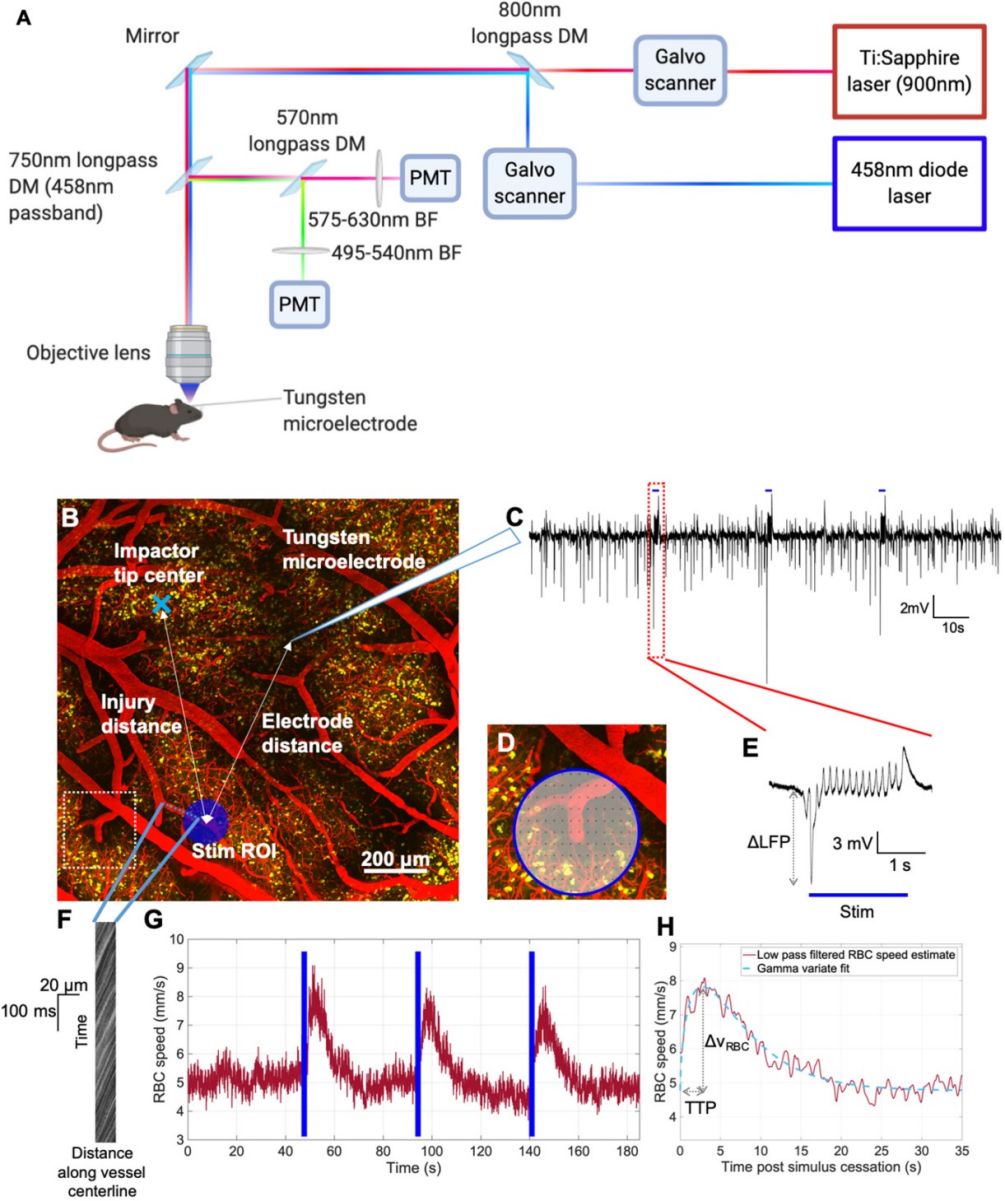
***In vivo* experimental paradigm for focused photostimulation and combined 2PFM/electrophysiological recording.** A. Lightpath diagram of imaging/stimulation configuration in the FVMPE-RS system (Olympus, Japan). DM: dichroic mirror. BF: barrier filter. PMT: photomultiplier tube. B. Maximum intensity projection of a 2PFM image stack over 500 μm of cortical depth, with the image acquired using 900 nm excitation wavelength with a 10x/0.5 NA water immersion objective (Olympus, Japan) at 1024x1024 matrix size (1.243 μm/pixel). Red: intravenous Texas Red (Invitrogen, USA). Yellow: ChR2-eYFP in pyramidal neurons. Light blue “x” denotes impactor tip centre, while dark blue circle indicates raster-scanned focused photostimulation ROI, with light blue dots representing the imaging linescan trajectory inside the vessel. The inset in D corresponds to the dotted white square in B. C. Representative trace of LFP recordings. Blue bars indicate focused photostimulation periods. D. Representative pixel trajectory for focused photostimulation presentation. E. LFP response to focused photostimulation. Blue bar indicates focused photostimulation period. F. Example XT image collected in a penetrating venule. G. v_RBC_ estimation via LSPIV method 46. Blue bars denote photostimulation periods. H. Low pass-filtered v_RBC_ response to focused photostimulation (red) and the corresponding gamma variate fit (light blue). Images in panel A generated via BioRender.com.

Two-photon fluorescence microscopy was performed following established methods 40. 2PFM was performed on an FVMPE-RS multiphoton microscope (Olympus, Japan) with 25x/1.05 NA or 10x/0.6 NA objective lenses (Olympus, Japan) for imaging and imaging/electrophysiology experiments, respectively. The mouse was positioned under the microscope and an Insight Ti:Sapphire laser (SpectraPhysics, USA) was used to excite YFP-labelled ChR2-expressing neurons and Texas Red-labelled vasculature at 900 nm. Emitted signals were acquired by photomultiplier tubes (PMTs) preceded by 485-540 nm or 575-630 nm barrier filters, separated by a 570 nm dichroic mirror (Chroma Technology, USA). Images down to cortical depths of 500-600 μm were captured prior to functional imaging (Figure 2). Thereafter, isofluorane was discontinued and anesthesia switched to alpha-chloralose (75 mg/kg induction, 40 mg/kg/hr maintenance) via an infusion pump (Harvard Apparatus, USA). The objective was positioned over the injury focus, and cortical penetrating vessels within that 2PFM imaging FOV (508 x 508 μm for the 25x objective, 1272 x 1272 μm for the 10x objective) were interrogated. The red blood cell (RBC) speed in these vessels was recorded through galvanometer-driven line scans (1.1-1.3 ms/line, 2 μs/pixel) along the longitudinal axes of these vessels, at the level of pre-diving point segments parallel to the cortical surface. The 458 nm (0.5 mW/mm^2^) photostimulation was raster scanned with a separate galvanometer over a 120 μm-diameter circular ROI centered over each penetrating vessel with 144-502 ms repetition period, 4 μs/pixel, 2-2.1 s duration, so as to stimulate neuronal soma and processes proximal to the imaged vessel while maintaining >70% of the photostimulated area extravascular. Photostimulation power was measured via a power meter (Model 842-PE, Newport, USA). Power density conversion was performed using the area of the stimulation ROI (120 μm diameter circular ROI).

### Local field potential recordings and data analysis

In a subset of experiments, local field potentials (LFPs) were recorded simultaneously with RBC speed. Mice were prepared as described above, but the cranial window was covered using PDMS instead of glass so as to allow for tungsten electrodes to be positioned in the cortical tissue below the cranial window 44, at a cortical depth of ca. 50 μm. LFP activity was captured via tungsten electrode mounted on a multi-manipulator (Scientifica, UK) and recorded through a patch-clamp amplifier operating in current clamp mode (MultiClamp 700B, Molecular Devices, USA) with an analog 20 Hz low-pass filter used to decrease background noise and enable real-time response visualization; and subsequently digitized for computer acquisition (Digidata 1440A, Molecular Devices, USA). Recordings were low pass-filtered post-hoc and LFP response parameters were extracted aparametrically. Extracted response parameters were peak LFP magnitude (ΔLFP) and area under the curve (AUC) during stimulus; as well as peak LFP magnitude and AUC following stimulus offset. To characterize neuronal activity at rest, power spectra of 20 s pre-stimulus baseline segments were acquired via FFT and divided into frequency bands as previously described 45. ΔLFP increased with decreasing distance between the region of the photostimulation and the location of the recording electrode tip. This effect lead us to scale ΔLFP, on a subject-wise basis, to the level observed at the mean electrode-to-photostimulation ROI distance of all mice (Figure 2B): all LFP readouts depicted were linearly corrected in this way, as shown in Figure S1.

### RBC speed data analysis

RBC speed was estimated from line scans as described earlier 46, allowing for robust speed estimation of fast-moving RBCs commonly seen in cortical penetrating arterioles. After image normalization and DC offset correction, sequential line scans were Fourier transformed, one FFT was conjugated and multiplied by the FFT of the other, and the inverse FFT was performed on the result to compute their cross-correlation. Gaussian peak-fitting was performed to calculate the spatial shift between adjacent line scans. RBC speed was calculated from this spatial shift while taking into account the line scan acquisition rate. RBC speed traces were then low-pass filtered and a 10-point moving average was applied to minimize noise and effects of respiratory/cardiac fluctuations. RBC traces following each stimulus presentation were fitted to a gamma function to estimate response magnitudes (Δv_RBC_), time to peak (TTP), full-width-at-half-maximum (FWHM), and area under the curve (AUC).

### Paired neurovascular measurements and neurovascular coupling ratio

In mice that underwent combined electrophysiology and 2PFM experiments, simultaneous neuronal and vascular recordings were made at rest and in response to photostimulation. At rest, Θ band power, acquired from spectral characterization of LFP baseline, and baseline RBC speed (v_base_) were linearly regressed. In the evoked state, the neurovascular coupling (NVC) ratio was calculated as the quotient of venular Δv_RBC_ AUC to ΔLFP AUC.

### Histological characterization

At experimental endpoint peripheral blood was flushed with phosphate buffered saline (PBS; 137 mM NaCl, 2.7 mM KCl, 10 mM Na_2_HPO_4_, 1.8 mM KH_2_PO_4_; pH 7.4) containing 0.1% heparin (1 i.u./mL; LEO Pharma Inc, Thornhill, ON) followed by 4% paraformaldehyde (PFA) (w/v) in PBS. Brains were post-fixed in 4% PFA overnight followed by cryoprotection in 30% sucrose (w/v) in PBS at 4°C. Brains were sectioned at a thickness of 40 µm via sliding freezing microtome (Leica Biosystems, Wetzlar, Germany). Brain slices were imaged on a Zeiss Observer Z1 microscope and images were analyzed via custom analysis in ImageJ. LaminB1 fluorescence was normalized on a slicewise basis to DAPI co-stain fluorescence to account for intersubject variance in staining. (Figure 7C). ɣH2AX expression was calculated from the fluorescence within neuronal nuclei, as determined by co-expression of DAPI and NeuN, and expressed as a percentage of area of those nuclei (Figure 7F). GFAP fluorescence was summed across images and normalized to the number of slices per image.

### Glial fibrillary acidic protein (GFAP) immunofluorescent staining

Forty-micron coronal sections were collected with a sliding microtome. 2 sections per animal at 160 µm apart were selected around the injury site (Bregma + 0.5 mm) for immunofluorescent staining. Tissue sections were washed with PBS (3 x 10 min) followed by an incubation in a blocking buffer (PBS with 0.2% triton-X-100, 0.2% bovine serum albumin (BSA) and 2% donkey serum) for 1 h at room temperature. Sections were incubated with rabbit polyclonal antibody against GFAP (Z0334, 1:500; DAKO) in blocking buffer overnight at 4°C. Alexa Fluor 647 Donkey anti-Rabbit secondary antibody (A31573, 1:200; Invitrogen) was incubated with DAPI (NucBlue Fixed Cell ReadyProbes Reagent, 2 drops/mL; Thermo Fisher) in PBS with 0.2% triton-X-100, 0.2% BSA for 2 h at RT. Sections were washed with PBS (3 x 10 min) and then mounted on VistaVision HistoBond slides (VWR) with polyvinyl-alcohol mounting medium with DABCO (PVA-DABCO, Sigma) and sealed with a coverslip.

### Lamin-B1 immunofluorescent staining

Free-floating tissue sections underwent antigen retrieval (10 mM Sodium Citrate buffer @ 81°C, 20 min) followed by incubation in a blocking buffer (PBS with 0.3% triton-X-100 and 5% serum) for 1 h at room temperature. Sections were incubated with rabbit polyclonal antibody against Lamin B1 (ab16048, 1:1000; abcam, Cambridge, MA) in blocking buffer overnight at 4°C. AlexaFluor Gt anti-Rb secondary antibody (A-11037, 1:200; Thermo Fisher, Waltham, MA) was incubated with DAPI (NucBlue Fixed Cell ReadyProbes Reagent, 2 drops/mL as per manufacturer's instructions; Thermo Fisher) in PBS with 0.1% triton and 0.5% bovine serum albumin (BSA) for 2 h at RT. Tissue sections were washed with PBS between each step (3 x 10 min). Tissue sections were then mounted on VistaVision HistoBond slides (VWR, Mississauga, ON) with a polyvinyl-alcohol mounting medium with DABCO (PVA-DABCO, Sigma, Oakville, ON) and sealed with a coverslip. N_mice_ = 23 (6 sham/vehicle, 5 sham/L-655,708, 6 TBI/vehicle, 6 TBI/L-655,708), N_images_ = 184 (4 slices/subject, 2 contra/ipsi images/slice).

### ɣH2AX/NeuN immunofluorescent staining

Free-floating tissue sections underwent antigen retrieval (0.25 mM EDTA at 95°C, 40 min), were allowed to cool (10 min, benchtop), and were then incubated in blocking buffer (Tris Buffered Saline (TBS) with 4% BSA, 0.02% tween-20, and 2% serum) for 1 h at RT. Sections were incubated with primary antibodies against phospho-Histone H2A.X and NeuN (mouse anti-H2A.X (05-636, 1:200), guinea pig anti-NeuN (ABN90, 1:500); both Sigma-Aldrich) in blocking buffer overnight at 4°C. AlexaFluor-labeled secondary antibodies (Dk-anti-Mo-594, A21203, Thermo Fisher; Dk-anti-GP-647, AP193SA6, Sigma) were diluted at 1:200 in TBS with 4% BSA and 0.02% tween-20 and incubated for 2 h at RT. Tissue sections were washed with TBS between each step (3 x 10 min). Tissue sections were then mounted on VistaVision HistoBond slides (VWR) with PVA-DABCO (Sigma) and sealed with a coverslip. N_mice_ = 20 (4 sham/vehicle, 4 sham/L-655,708, 5 TBI/vehicle, 7 TBI/L-655,708), N_images_ = 160 (4 slices/subject, 2 contra/ipsi images/slice).

### Statistical analysis

The effects of injury and treatment were assessed by generalised linear modelling using the lme4, lmerTest, and nlme packages in R, with subjects treated as a random effect. This modelling accounts for variation within and between subjects to yield robust estimates in the presence of unbalanced group sizes. Fixed effects in linear mixed effect models were condition (injury/sham), treatment (L-655,708/vehicle), and, for histological data, hemisphere (contra-/ipsilateral to injury). Interaction terms between fixed effects were included in the modelling. The contrast across condition and treatment groups of the different readouts was thus modeled as:


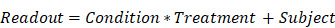


where multiplication in this notation denotes both the main effects and interaction between the two terms. Since the LFP magnitude estimates were not normal, they were log transformed prior to linear modelling. Sex was included as a fixed effect in preliminary analysis but was not found to exert a significant effect on any readout and was thus not made a part of the model in the final analysis.

## Results

### Resting venular RBC speed and neuronal Θ power are altered in mTBI mice

**Figure 3:**
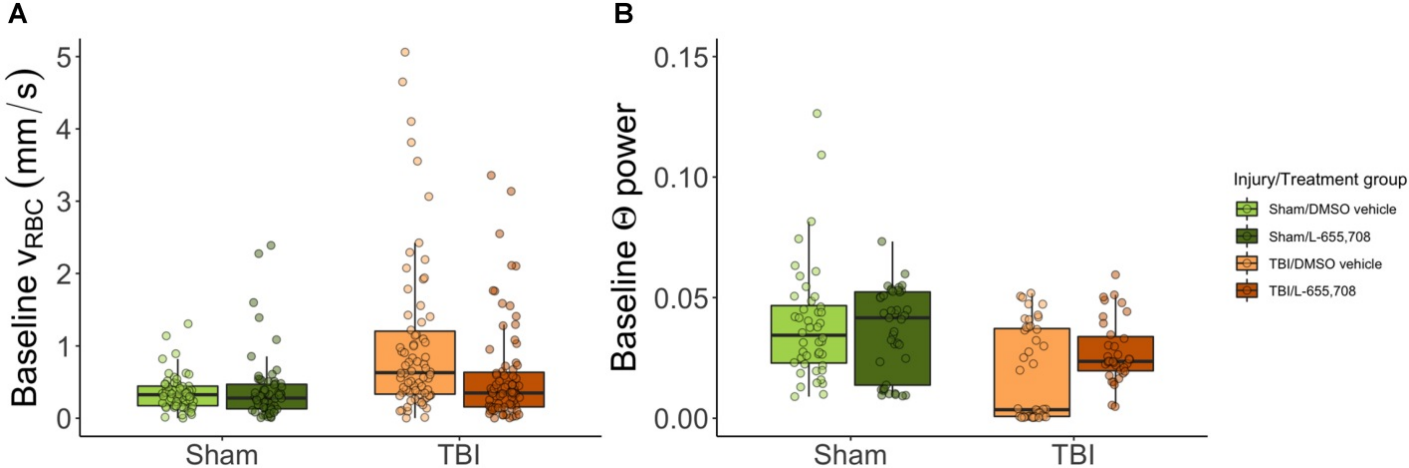
** Baseline RBC speeds in penetrating venules and baseline neuronal Θ power are elevated in mTBI mice but normalized to sham levels in L-655,708 treated mTBI animals.** A-B. Points indicate the mean of pre-stimulus baseline v_RBC_ across 3 photostimulation presentations on a single vessel. A. Pre-stimulus baseline v_RBC_ in penetrating venules. Baseline venular speeds are elevated in vehicle-treated mTBI mice, but indistinguishable from sham levels in L-655,708-treated mTBI mice. Δv_base injury_ = 115 ± 25%, p_injury_ = 0.000005, p_treatment_ = 0.78, Δv_base injury x treatment_ = -88 ± 35%, p_injury x treatment_ = 0.01. N_Sham/DMSO vehicle_ = 6, N_Sham/L-655,708_ = 6, N_TBI/DMSO vehicle_ = 10, N_TBI/L-655,708_ = 7. B. Neuronal Θ (4-8 Hz) power recorded during venular linescanning shown in A. mTBI induced an attenuation in Θ power, with a trend toward amelioration of this effect in L-655,708 treated cohort. ΔΘ_injury_ = -57 ± 79% p_injury_ = 0.01, p_treatment_ = 0.93, ΔΘ_injury x treatment_ = 27 ± 59%, p_injury x treatment_ = 0.08. N_Sham/DMSO vehicle_ = 5, N_Sham/L-655,708_ = 5, N_TBI/DMSO vehicle_ = 5, N_TBI/L-655,708_ = 5.

We first examined the resting hemodynamics by comparing pre-stimulus v_RBC_ (v_base_) across groups. At rest, arterioles did not exhibit any baseline RBC speed contrast. In contrast, RBC speed in penetrating venules of mTBI mice was twice that of sham animals (Δv_base injury_ = 115 ± 25%, p_injury_ = 0.000005, p_treatment_ = 0.78). Such increased venular baseline RBC speeds were not present in mTBI mice treated with L-655,708 (Δv_base_ = -88 ± 35%, p_injury x treatment_ = 0.01) as displayed in Figure 3A. Resting vessel diameters were not found to be significantly different across groups aside from a trend toward venular dilation due to mTBI (Δd_venule_ = 14.5 ± 9.1%, p_venule injury_ = 0.1). The standard deviation of venular baseline v_RBC_ (σ_base_) was increased in mTBI (Δσ_base_= 60 ± 39%, p_injury_ = 0.0002, p_treatment_ = 0.54, p_injury x treatment_ = 0.07, Figure S3D). There was, however, no difference in the corresponding coefficient of variation (COV = σ_base_/v_base_), indicating that the contrast in standard deviation resulted from the proportionate increase in v_base_ (Figure 3B). Analysis of power spectra of the baseline LFP recordings indicated that the theta band (4-8 Hz 45) neuronal power was reduced in mTBI mice (Figure 3B, ΔΘ_injury_ = -57 ± 79%, p_injury_ = 0.01). We also observed a trend toward a reduced effect of injury on neuronal theta band power in L-655,708 treated mTBI mice (ΔΘ_injury x treatment_ = 27 ± 59%, p_injury x treatment_ = 0.08).

### Reduced neuronal reactivity in mTBI mice

**Figure 4:**
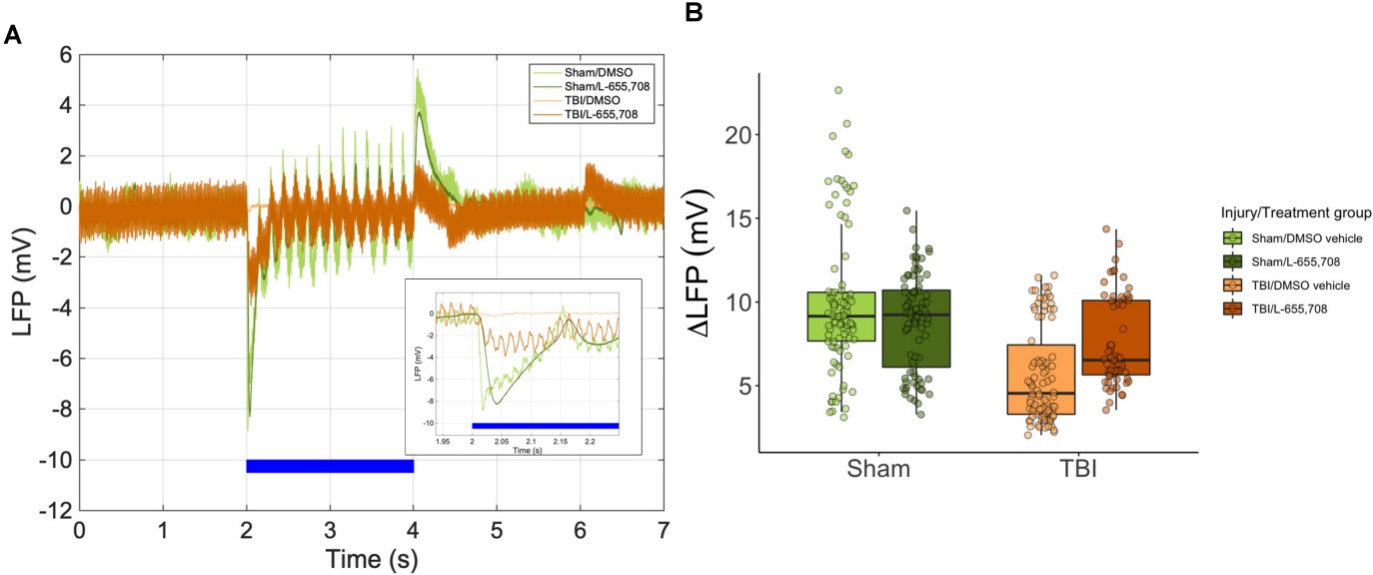
** Neuronal hyporeactivity in mTBI mice is partially normalized in L-655,708 treated mTBI animals.** A. Representative LFP traces during photostimulation. Inset depicts the early response. Blue bar indicates the photostimulation period. B. Peak magnitude of electrode distance-corrected LFP response to focused photostimulation. Lower responses were seen in vehicle-treated mTBI mice (ΔLFP = -47 ± 28%, p= 0.018), and a simplified linear model indicated that Sham/L-655,708 and TBI/L-655,708 groups were indistinguishable (p = 0.59). Points indicate the mean LFP peak across 3 stimulus presentations on each vessel. N_Sham/DMSO vehicle_ = 5, N_Sham/L-655,708_ = 5, N_TBI/DMSO vehicle_ = 5, N_TBI/L-655,708_ = 5.

To investigate changes in local neuronal activity and neurovascular unit function post-injury, 2PFM measurements of vascular reactivity were paired with local field potential recordings via a tungsten microelectrode 44. ΔLFP was attenuated in the mTBI group (ΔLFP = -47 ± 28%, p = 0.018), as shown in Figure 4. A simplified linear model further showed that there was no statistically significant difference between sham and mTBI L-655,708-treated mice (p = 0.59). Of note, as shown in Figure 2B, the Euclidean distance between photostimulation ROI/vessel diving point and the injury focus was recorded: however, no significant correlation between this distance and any neuronal measures were observed within the 1272 x 1272 μm imaging FOV used here. These findings show that mTBI may elicit sustained reduction in neuronal reactivity and that low-dose L-655,708 intervention can prevent this effect.

### Augmented venular reactivity in mTBI mice

**Figure 5:**
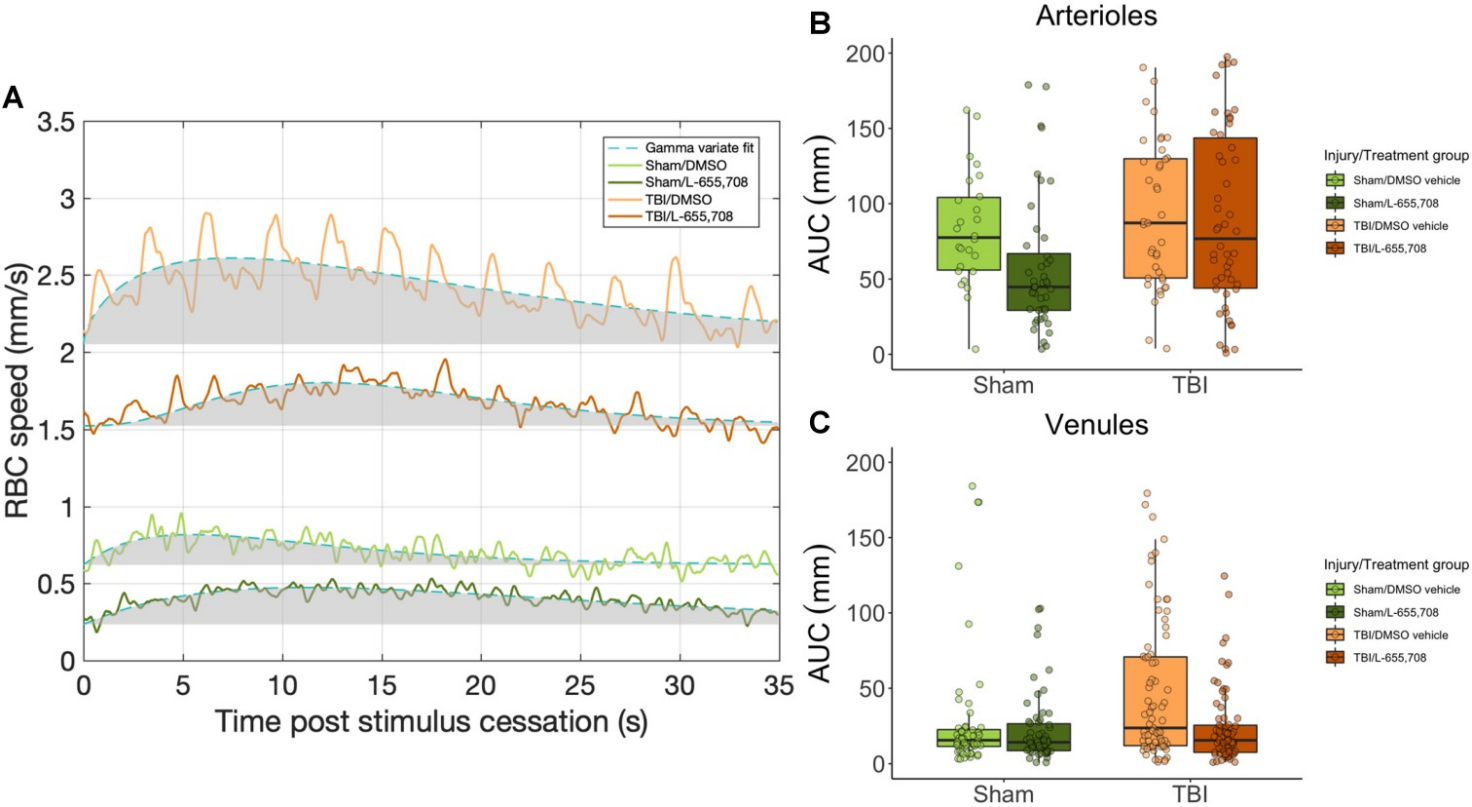
** Venular hyperreactivity in mTBI mice is normalized to sham levels following L-655,708 administration.** A. Venular v_RBC_ responses to focused photostimulation in representative animals from each cohort. Shaded grey area represents the AUC plotted across all animals in B. B. AUC of arteriolar responses to focused photostimulation. Each point represents the mean response AUC across 3 stimulus presentations on each vessel. C. AUC of venular responses to photostimulation was significantly elevated in vehicle-treated mTBI mice. Each point represents the mean response AUC across 3 stimulus presentations on each vessel. L-655,708 treatment prevented this elevation. ΔAUC_injury_ = 53 ± 17%, p_injury_ = 0.001, ΔAUC_injury x treatment_ = -66 ± 24%, p_injury x treatment_ = 0.005. N_Sham/DMSO vehicle_ = 6, N_Sham/L-655,708_ = 6, N_TBI/DMSO vehicle_ = 10, N_TBI/L-655,708_ = 7.

Vascular responsivity was evaluated in the chronic stage of repeated mTBI by measuring single cortical penetrating vessel responses to focused photostimulation (Figure 5). Area under the curve (AUC) of arteriolar responses to focused photostimulation was found to be indistinguishable between cohorts (Figure 5B), whereas venular reactivity was elevated in mTBI mice (Figure 5C, ΔAUC = 53 ± 17%, p = 0.001). This venular hyperreactivity was prevented by L-655,708 treatment (ΔAUC = -66 ± 24%, p = 0.005). Our findings here indicated sustained venular hyperreactivity post mTBI and a potential for its prevention by an early, low dose L-655,708 intervention. Similarly to neuronal recordings, no significant correlations between distance from injury focus and any vascular measures were observed within our 508 x 508 μm imaging FOV.

### Neurovascular coupling is altered in mTBI mice

**Figure 6:**
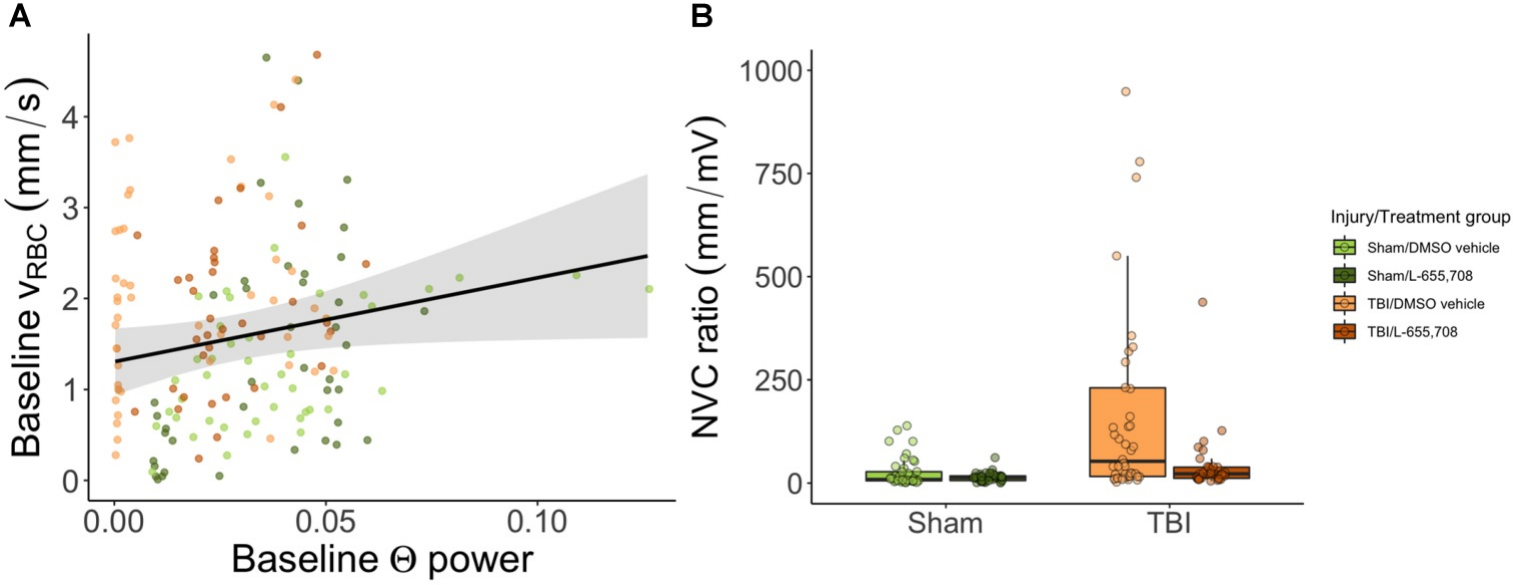
** Neurovascular coupling.** A. Linear regression of baseline v_RBC_ of all penetrating venules versus baseline theta power based on electrophysiological data acquired via the electrode in the imaging field of view. There was a significant positive linear dependence of baseline v_RBC_ on baseline Θ power (p = 0.009, slope = 9 ± 4), but no dependence on injury (p = 0.89) or treatment (p = 0.44). Solid line represents a linear mixed effects model fit of all groups. B. Neurovascular coupling (NVC) ratio was calculated as the ratio of vascular response AUC to paired neuronal response AUC. Shown here are venular NVC ratios depicting an increased NVC in mTBI vehicle mice (ΔNVC = 674 ± 304%, p = 0.039), with L-655,708-treated sham and L-655,708-treated mTBI groups being indistinguishable (p = 0.88). N_Sham/DMSO vehicle_ = 5, N_Sham/L-655,708_ = 5, N_TBI/DMSO vehicle_ = 5, N_TBI/L-655,708_ = 5.

Lastly, we examined the relationship between neuronal and vascular reactivity to test for changes to neurovascular coupling. Paired neuronal and venular function measurements at rest were linearly regressed to assess the effects of mTBI on resting state (Figure 6). We observed a significant dependence (p = 0.009, slope = 9 ± 4) of the baseline venular v_RBC_ on neuronal theta power at baseline (Figure 6A), with no effect of either injury or treatment. For responses to photostimulation, the neurovascular coupling (NVC) ratio was calculated as the ratio of vascular to neuronal AUC following photostimulation. We observed a significant increase in NVC ratio due to mTBI as shown in Figure 6B (ΔNVC = 674 ± 304%, p = 0.039) and further that mTBI mice treated with L-655,708 were indistinguishable from their uninjured counterparts (p = 0.88), implying an increased venular NVC component in the injured state that may be prevented through GABAAR inverse agonism.

### Reduced LaminB1 expression and increased ɣH2AX expression in mTBI mice

**Figure 7:**
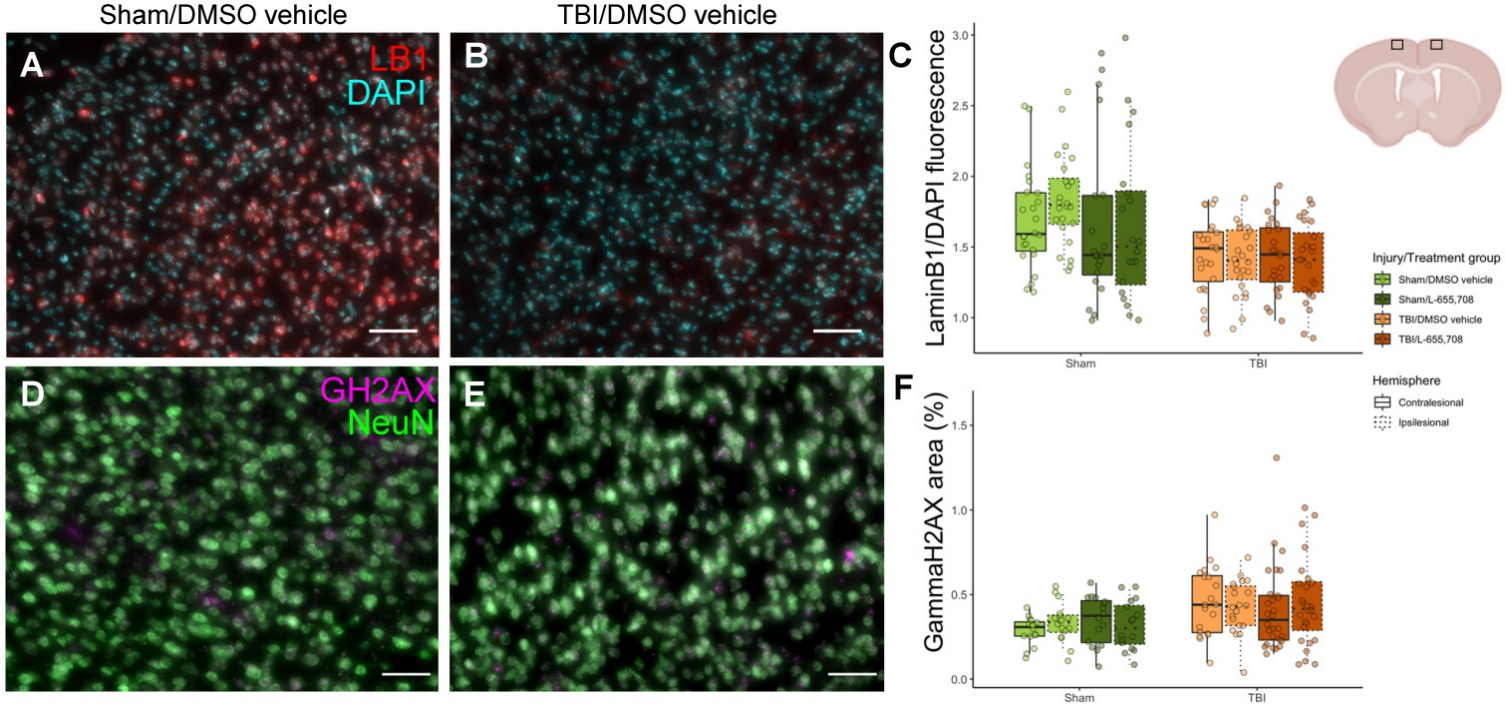
** Histological characterization of the mTBI model.** A. DAPI (cyan) /LaminB1 (red) staining of a brain slice maximum intensity projection along the A-P dimension in the ipsi-contusional hemisphere of a vehicle-treated sham mouse at the sham impact coordinates. B. DAPI/LaminB1 staining of a brain slice maximum intensity projection along the A-P dimension in the right hemisphere of an mTBI mouse at the impact coordinates. Scale bar = 50 μm. C. Total LaminB1 fluorescence normalized by total DAPI fluorescence in each image slice across injury/treatment groups. LaminB1 fluorescence is significantly reduced in mTBI mice (ΔLaminB1/DAPI = -17 ± 8%, p_injury_ = 0.047). N_mice_ = 23 (6 sham/vehicle, 5 sham/L-655,708, 6 TBI/vehicle, 6 TBI/L-655,708), N_images_ = 184 (4 slices/subject, 2 contra/ipsi images/slice). Representation of imaging locations on the brain slices. Image from biorender.com. D. NeuN (green) /ɣH2AX (magenta) staining of a brain slice maximum intensity projection along the A-P dimension in the right hemisphere of a vehicle-treated sham mouse at the sham impact coordinates. E. NeuN/ɣH2AX staining of a brain slice maximum intensity projection along the A-P dimension in the right hemisphere of an mTBI mouse at the impact coordinates. Scale bar = 50 μm. F. Fractional area of ɣH2AX expression within neuronal nuclei in each image slice across injury/treatment groups. ɣH2AX expression is significantly increased in mTBI mice (ΔɣH2AX = 43 ± 13%, p_injury_ = 0.011). N_mice_ = 20 (4 sham/vehicle, 4 sham/L-655,708, 5 TBI/vehicle, 7 TBI/L-655,708), N_images_ = 160 (4 slices/subject, 2 contra/ipsi images/slice).

To independently assess the mTBI sequelae, we evaluated several histological markers in the pericontusional tissue. Neuronal survival (on NeuN) and glial activation (on GFAP) were indistinguishable between mTBI and sham cohorts. We next evaluated laminB1 and ɣH2AX as prior clinical and preclinical studies reported on TBI-elicited senescence, manifested as nuclear membrane loss (and thus reduced cerebral laminB1 expression) 47, 48, and DNA damage (and hence increased ɣH2AX expression in neuronal nuclei) 47. LaminB1 expression was visibly reduced in mTBI vs. sham mice as shown in Figure 7C (ΔLaminB1/DAPI = -17 ± 8%, p_injury_ = 0.047). ɣH2AX expression was increased in mTBI cohort, as demonstrated in Figure 7F (ΔɣH2AX = 43 ± 13%, p_injury_ = 0.011). YFP expression in brain slices was also quantified to test whether ChR2 expression were altered following mTBI; however, no such changes were observed (Figure S4). No significant differences were observed between ipsi- and contracontusional hemispheres in mTBI mice in any histological readout (p_LaminB1 hemisphere_ = 0.25, p_ɣH2AX hemisphere_ = 0.84).

## Discussion

The cellular level changes elicited by mild traumatic brain injury are not well understood 49-51. Previous preclinical mTBI studies have largely focused on structural changes and/or mesoscopic scale functional measures 21, 24, 25, 31, 52-56. Here, for the first time, we paired single vessel measurements with intracortical recordings and applied focal photostimulation to elucidate neurovascular unit dysfunction post mTBI. We demonstrated beneficial effects of delayed, chronic, low dose disinhibition, via GABAAR inverse agonism, on the neurovascular function. Given the importance of neurovascular physiology for brain health 17, our findings motivate the development of post-concussion interventions that target the neurovascular unit to promote long term resilience of the concussed brain. Successful application of such interventions in heterogeneous clinical populations will be predicated on the development of translational biomarkers of neurovascular dysfunction post mTBI, thereby allowing optimization of treatment to individual patients' needs.

The impact parameters in this model were set within previously-described guidelines for inducing mild injury with the controlled cortical impact 57, 58. The resulting injury was designated as 'mild' given lack of contrast on structural MRI, the criterion commonly applied in clinical practice 13-15. That the injury produced was mild was further supported by the lack of group contrast on both NeuN and GFAP staining. While microhemorrhages, dural damage, and vessel rupture are common occurrences in the acute stage of mTBI 59-62, they are expected to resolve rapidly and were indeed not presently observed at two weeks post final insult. Prior preclinical studies have characterized TBI pathophysiology via morphological, functional, and behavioural assays 7-9, 19, 20, 22, 24-26, 30, 31, 50-52, 54-56, 63-71. The presence and extent of these changes has varied widely across studies depending on the age of subject 30, 65, 72, time post-injury 19, 31, 54, 63, 64, 70, number of injuries 30, 31, and injury model 65, 67. In the case of mild TBI, it has been shown that long-term changes may arise in both patients and in rodent models from either single or repeated injuries 11, 49. Consistent with this, we observed neurovascular impairments two weeks post third impact. The current study used laminB1 and ɣH2AX expression as markers of senescence in the absence of cohort-wise differences with respect to either NeuN or GFAP expression. Dramatic laminB1 expression reductions, which are associated with nuclear membrane ruptures and cell death 73, have been observed in TBI and in preclinical models of TBI 47, 72. In the present mild TBI model, a limited decrease of laminB1 expression was observed as well. Furthermore, heightened ɣH2AX expression, indicative of DNA damage, has been reported post mortem in TBI brains 47, a feature recapitulated by our model. Of note, these effects were not specific to the impacted hemisphere, indicating that closed head impacts elicit bilateral damage and/or that the injury is relayed by the two-week time point. Acute bilateral damage is likely a result of contrecoup injury 61, with the impactor causing deformation of the impacted cortex and contusion of the contralateral cortex. This is in line with cortical impact models being described as “mainly focal” 57. Neuronal survival and astrogliosis, as measured by NeuN and GFAP expression, were indistinguishable among groups. In a similar model, we have previously reported no NeuN contrast alongside ipsicontusional GFAP contrast 25, as with earlier work 31, 74; other studies, in contrast, did find reductions in neuronal survival 75-77. Despite the lack of group contrast, gliosis was visible in some of our mTBI mice.

The cerebrovascular changes observed in mTBI patients in the chronic stage post trauma exhibit high variance with respect to presence, directionality, and magnitude of cerebral blood flow (CBF) signal changes 7, 13, 20, 25, 50-54, 68, 78. The majority of studies have reported a reduction in baseline arterial blood flow 13, 52, 78 and an attenuation of stimulation-elicited cerebrovascular responses measured 6+ months post mTBI 13, 52. Several studies have reported decreases 79-82 in CBF in the weeks to months following mTBI via ASL fMRI, whereas others have shown increases 78, 83-85, no change 86-89, or a combination of spatially-dependent changes 90-93 in this timeframe. In preclinical models of mTBI, hypoperfusion has been observed at 1-4 weeks post TBI 25, 70 and within 1 hour to 1 day of injury 19, 22, 24. Similar to the clinical data, findings in preclinical models are highly heterogeneous but widely report CBF and cerebrovascular reactivity reductions post-injury 38, 94, whereas we presently report venular hyperreactivity, analogous to previous clinical findings 13, 78 and the first preclinical study to report this to our knowledge. While conventional readouts may be valuable for establishing the presence and severity of injury, quantitative functional measurements at the single-unit level are necessary for an in-depth understanding of the neurovascular sequelae of TBI pathophysiology 8, 9, 49, 50, 56 at a single-unit level 49, 50, and for establishing the absolute magnitude of these changes 13, 95. Here, we observed doubling of venular baseline RBC velocities, a trend toward venular dilatation of about 15%, as well as a ~50% elevation in venular reactivity. Analogously, earlier work in mTBI patients reported increased CBF using BOLD and ASL fMRI 5-6 weeks 83 and 6+ months 52, 56, 68, 78, 87 post injury. Evaluation of BOLD fMRI responses to working memory task in mTBI patients 4+ months post-injury has revealed attenuated responses of peri-contusional vasculature 87, whereas other studies using hypercapnic challenge with BOLD fMRI have shown increased reactivity 2+ weeks following injury 89 and with ASL 1 week following injury 88. The presently-observed venular specificity of the treatment effect on the brain vasculature may be due to venular measures reflecting an integration of all upstream changes in the microvascular network 96 capillaries are likely affected by trauma as well 21, 97, acquiring a representative sample size of these numerous microvessels would have taken prohibitively long. Additionally, the intrinsically slow nature of laser scanning (vs. relatively high speed of RBCs in cortical penetrating vessels) prevented us from making robust measurements of photostimulation-induced changes in the vessel diameter concomitant to the vessel's vRBC estimation.

Neurovascular coupling was interrogated in this study through ratios of paired neuronal and venular measures. While resting coupling between neuronal and vascular function remain unaltered, the NVC ratio was increased by mTBI. This finding suggests an alteration of neurovascular signaling that may be an adverse sequela of mTBI or a compensatory effect to potentiate the support of the injured tissue. At rest, venular baseline v_RBC_ was linearly dependent on baseline neuronal theta power; this dependence was not affected by either injury or treatment. While gamma band neuronal power would have been interesting to evaluate due to its role in neurovascular coupling 98, such analysis was presently precluded by the 20 Hz low-pass analog filtering used to mitigate the noise in the electrophysiological recordings introduced by the concurrent microscopy and by the use of a tungsten electrode (to accommodate a bulky objective of concurrent 2PFM and electrophysiology recordings). On the whole, our observations support the notion of evoked response assays being more sensitive measures of brain (dys)function than the baseline state recordings 13.

Interneuron-mediated tonic inhibition has been previously implicated in preclinical mTBI pathophysiology 8, 9, 26, 27. L-655,708 distribution in the brain following i.p. administration of 3 mg/kg (vs. 2.4 mg delivered subcutaneously, over 12 days, in the current study) has previously been shown to result, within 1-2 h following injection, in widespread distribution across the neocortical tissue 99. In contrast to the neocortex, little L-655,708 was observed in the cerebellum and the subcortical regions. Excitatory/inhibitory signaling balance is disrupted in mTBI as well as in severe injuries 8, 9 and this imbalance is thought to cause hyperreactivity in the immediate aftermath of injury followed by hyporeactivity in the subacute phase of injury 9. A delayed interventional window may thus be present in the hyporeactive phase post-injury. In months following mTBI, it has been shown that functional changes mediated by GABAA receptor currents occur in absence of an increase in receptor count 27. Additionally, one week following mTBI, impaired behaviour and synaptic plasticity were previously prevented via administration of L-655,708 intraperitoneally 26. Here we demonstrate the neurophysiological effects of such treatment at the single-unit level *in vivo*: specifically, a prevention of venular deficits and a normalization of neuronal responses in the L-655,708 treated vs. vehicle administered mTBI animals. The venular normalization observed in mTBI/L-655,708 mice may have been secondary to the normalization of neuronal tone and/or induced by the decreased availability of GABA, which is itself vasoactive 100, 101. This indicates that a well-timed (subacute phase), sustained, low-dose GABAA receptor inverse agonism may have preventative effects on the neurovascular aberrations elicited by mTBI and identifies the excitation/inhibition signaling imbalance as an important mechanism of injury and hence treatment target. Furthermore, this study was performed with functional recordings *in vivo*, providing a greater translational potential than the previously reported *ex vivo* measures 8, 9, 26, 27. Of note, L-655,708 administration in this study was systemic (via a subcutaneous osmotic pump) and is thus easily translatable. The resulting widespread effect on the neocortex 99 may have been beneficial considering the aforementioned spatial propagation of injury.

In summary, the present study describes sustained neurovascular impairments in a preclinical model of repeated closed head injury. We showed that in addition to neuronal hyporeactivity (-47 ± 28%) and reduced activity at rest (-57 ± 79%), the pericontusional venular function is abnormally elevated at rest (115 ± 25%) and upon stimulation (53 ± 17%). Increased resting cerebral blood flow has been observed at a regional level via MRI in mTBI patients 13, 56, 68, 78 but has not been shown at the level of individual vessels. Elevated venular responses to neuronal activation is a novel finding in preclinical mTBI, with analogous pathology described clinically 13, 88, 89. Delayed, low dose GABAA receptor inverse agonism via L-655,708 26, 27, 34, 35 to alleviate hypothesized tonic inhibition post TBI 8, 9, 26, 27, 34, 35 prevented the mTBI-induced venular impairment and normalized the neuronal reactivity. Our study provides new insight into the neurophysiological sequelae of mTBI, constitutes a first step in the development of translational markers of neurovascular pathology in mild TBI that is silent on neuroimaging, and suggests benefits of attenuating tonic inhibition in the subacute to chronic stage of injury.

## Supplementary Material

Supplementary figures.

## Figures and Tables

**Figure 1:**
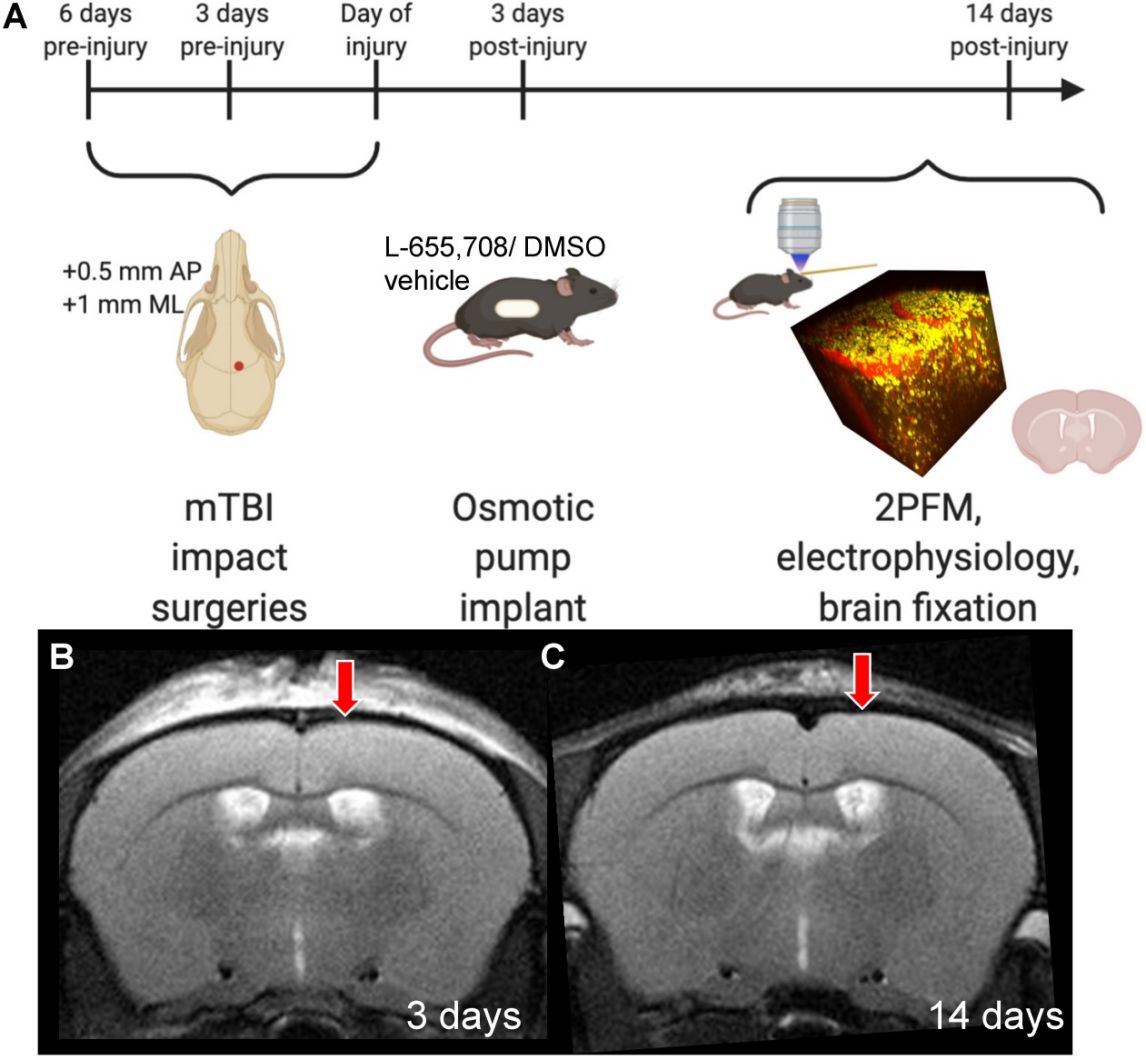
** Experimental timeline.** A. Mice received 3 mild impacts over the skull at the location indicated by the red dot (+0.5 mm AP, +1 mm ML) with a 3 day inter-impact interval. Impacts were delivered with a 1.5 mm impactor tip diameter, at 2 m/s, with a 200-ms dwell time. 3 days post-injury (DPI), mice were subcutaneously implanted with an osmotic pump containing either L-655,708 in saline/DMSO solution or vehicle alone. At 14 DPI, mice underwent 2PFM imaging and a subset of mice underwent concurrent local field potential recordings via intracortical tungsten microelectrode. A subset of mice brains did not undergo imaging, but were instead fixed for immunohistochemistry. B. Representative T2-weighted MR image in an injured mouse at 3 DPI. Red arrow indicates impact location. C. Representative T2-weighted MR image in an injured mouse at 14 DPI. Red arrow indicates impact location. Images in panel A were generated via BioRender.com.

**Table 1:** Summary of animals and vessel recordings across cohorts.

Group	2PFM Cohort		2PFM + EP Cohort	
	Mice	Vessels	Mice	Vessels
Sham/DMSO vehicle	6	34 arterioles 61 venules	5	50 arterioles 44 venules
Sham/L-655,708	6	49 arterioles 63 venules	5	43 arterioles 41 venules
TBI/DMSO vehicle	10	50 arterioles 82 venules	5	42 arterioles 46 venules
TBI/L-655,708	7	56 arterioles 79 venules	5	28 arterioles 35 venules

**Table 2:** Summary of physiological readouts of injury/treatment groups. No significant difference in any physiological parameters was seen across groups.

	Sham/DMSO vehicle	Sham/ L-655,708	TBI/DMSO vehicle	TBI/L-655,708
**End tidal CO2 (mmHg)**	15 ± 3	18 ± 5	19 ± 3	16 ± 3
**Body temperature (°C)**	37.1 ± 0.1	37.1 ± 0.6	37.1 ± 0.1	37.0 ± 0.2
**O2 saturation (%)**	97 ± 2	97 ± 3	98 ± 1	97.3 ± 0.6
**Heart rate (bpm)**	353 ± 61	338 ± 42	397 ± 102	376 ± 39
