# Supplementary material for: Attenuation of tonic inhibition prevents chronic neurovascular impairments in a Thy1-ChR2 mouse model of repeated, mild traumatic brain injury
Source: Theranostics. 2021 Jun 16;11(16):7685–99. doi: 10.7150/thno.60190 (PMC8315057; doi:10.7150/thno.60190)
Supplement: Supplementary file 1 — Supplementary figures. [file thnov11p7685s1.pdf]

Supplementary materials

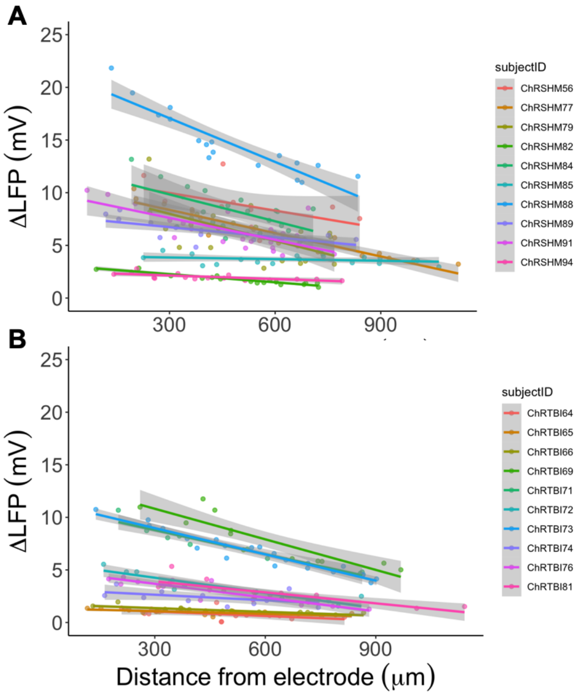

Supplementary Figure 1: LFP response magnitude versus focused photostimulation ROI distance from the LFP recording electrode. Subjectwise linear regressions of LFP responses to focused photostimulation versus distance of the photostimulation ROI from the electrode tip (Figure 2). A. Linear regressions for sham subjects. B. Linear regressions for TBI subjects.

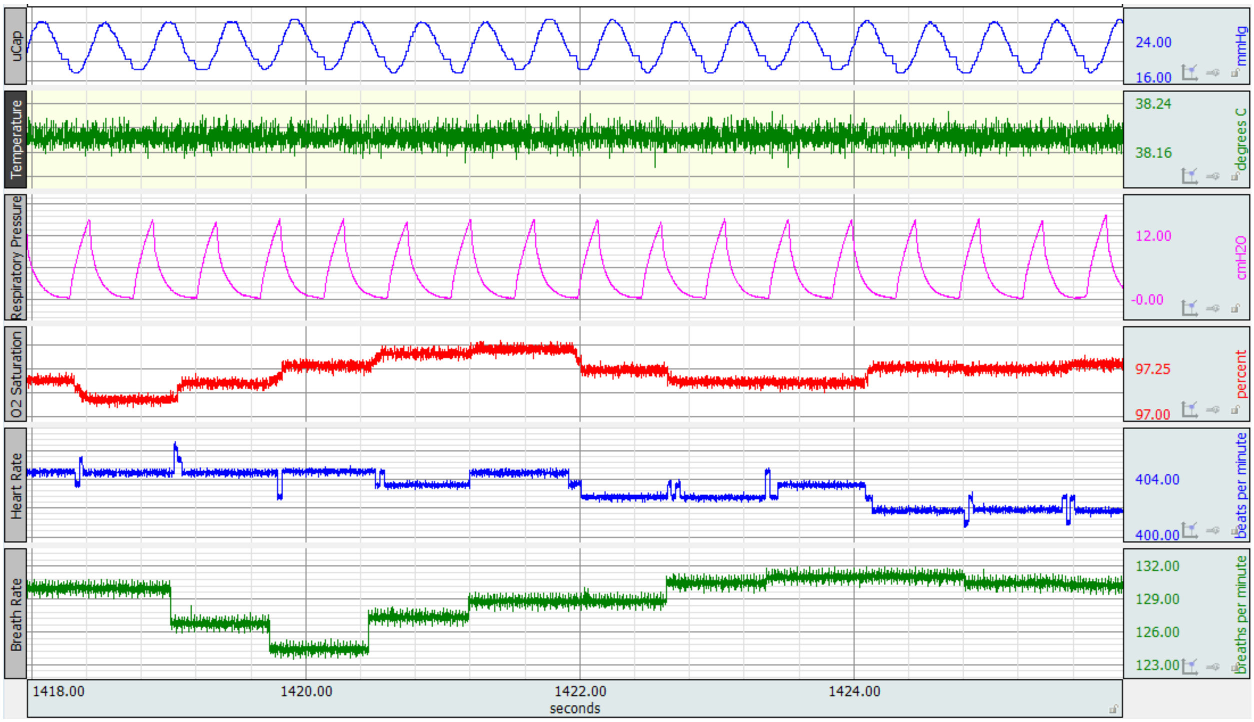

Supplementary Figure 2: Physiological monitoring data. Physiological monitoring data recorded during experiments (AcqKnowledge software, BIOPAC, Canada) included microcapnography (uCap) to monitor end-tidal CO2, temperature monitoring, respiratory pressure of mechanical ventilation, oxygen saturation of blood, heart rate, and breath rate. No differences in physiological monitoring between cohorts were observed.

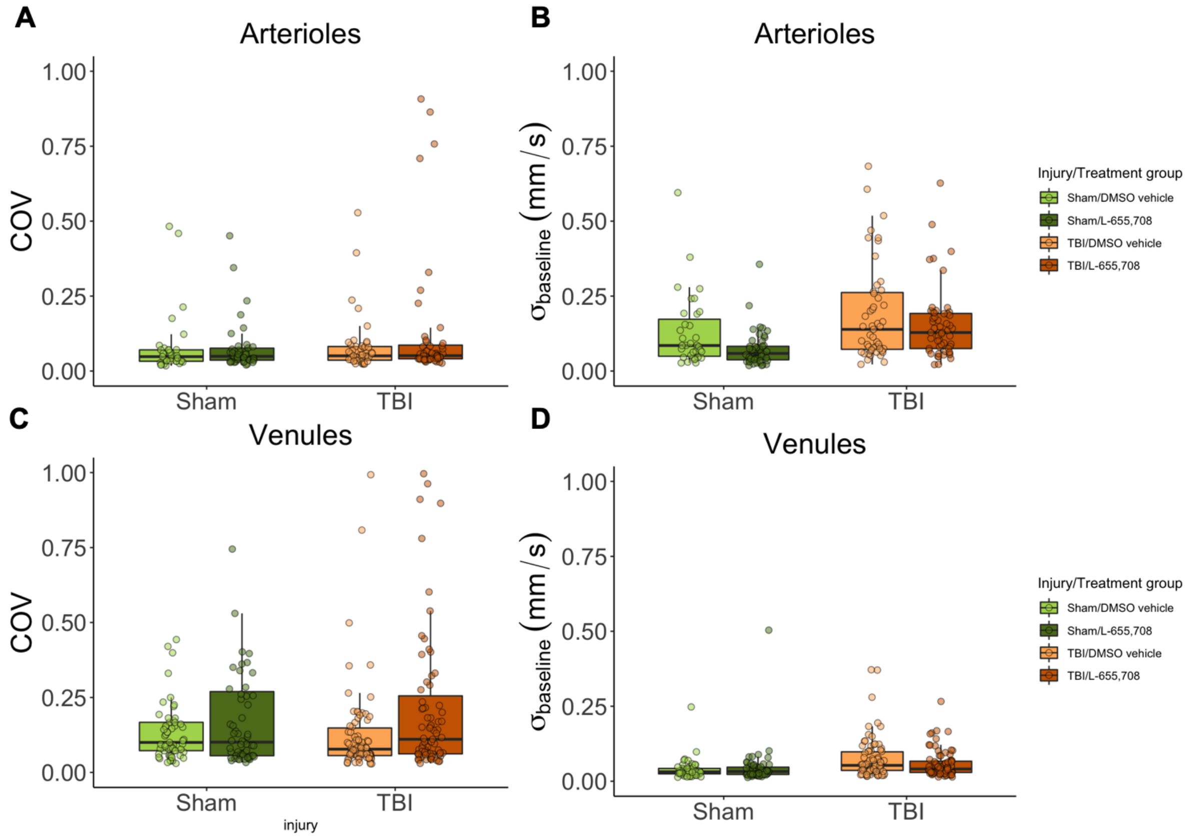

Supplementary Figure 3: Coefficient of variation (COV) and standard deviation (σ) of pre-stimulus baseline v_{RBC}. Measures were taken over 10s prior to stimulation. A. COV of baseline vRBC in arterioles (p_{injury} = 0.7, $p_{treatment}$ = 0.1, $p_{injury\ x\ treatment}$ = 0.09). B. σ_{base} of vRBC in arterioles (p_{injury} = 0.2, $p_{treatment}$ = 0.8, $p_{injury\ x\ treatment}$ = 0.6). C. COV of baseline vRBC in venules (p_{injury} = 0.7, $p_{treatment}$ = 0.7, $p_{injury\ x\ treatment}$ = 0.7). D. σ_{base} of vRBC in venules ($\Delta\sigma_{base\ injury}$ = 0.025 ± 0.006 mm/s, p_{injury} = 0.0002, $p_{treatment}$ = 0.5, $p_{injury\ x\ treatment}$ = 0.07). $N_{Sham/DMSO\ vehicle}$ = 6, $N_{Sham/L\text{-}655,708}$ = 6, $N_{TBI/DMSO\ vehicle}$ = 10, $N_{TBI/L\text{-}655,708}$ = 7.

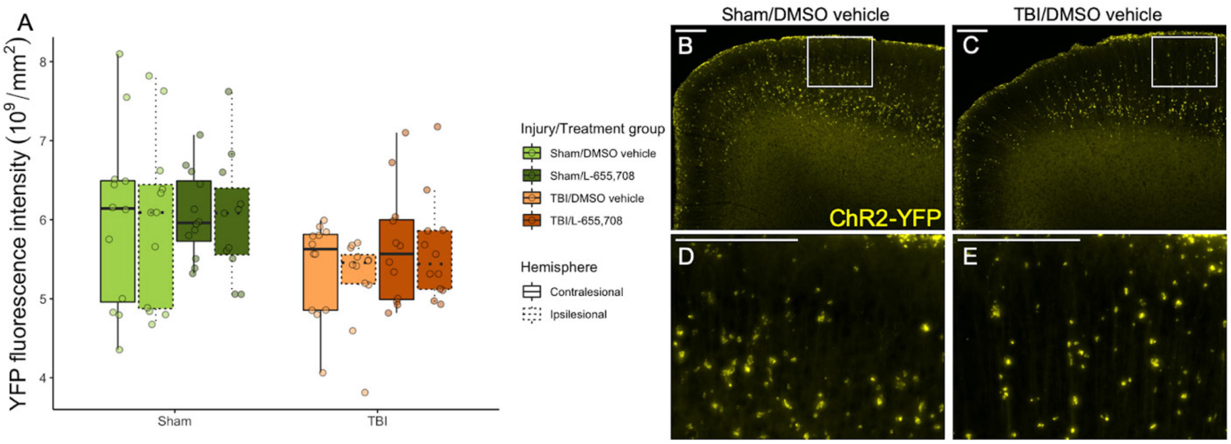

Supplementary Figure 4: ChR2-YFP expression is unchanged in mTBI mice. A. Sum of YFP fluorescence intensity in sections of the ipsicontusional hemispheres of mice across all groups. No significant changes across any groups were observed (p_{injury} = 0.2, $p_{treatment}$ = 0.9, $p_{hemisphere}$ = 0.8). N_{mice} = 6/group, N_{slices} = 4/mouse. B. Maximum intensity projection (along the A-P direction) of ChR2-YFP fluorescence in the ipsicontusional hemisphere of vehicle-treated sham mouse at the sham impact coordinates. White box indicates 20x inset in D. C. Maximum intensity projection (along the A-P direction) of ChR2-YFP fluorescence in the ipsi-contusional hemisphere of an mTBI mouse at the impact coordinates. White box indicates 20x inset in E. Scale bars = 200 µm.
